# Long-term work disability due to type I and II bipolar disorder: findings of a six-year prospective study

**DOI:** 10.1186/s40345-022-00264-6

**Published:** 2022-07-11

**Authors:** Petri Arvilommi, Sanna Pallaskorpi, Outi Linnaranta, Kirsi Suominen, Sami Leppämäki, Hanna Valtonen, Erkki Isometsä

**Affiliations:** 1 Department of Psychiatry, University of Helsinki and Helsinki University Hospital, Helsinki, Finland; 2 Finnish Institute for Health and Welfare, Helsinki, Finland; 3 Department of Mental Health and Substance Abuse, Social Services and Health Care, Helsinki, Finland

**Keywords:** Bipolar disorder, Cohort studies, Disability, Disability pension

## Abstract

**Background:**

Bipolar disorder (BD) is one of the leading causes of disability worldwide. However, the prevalence and predictors of long-term work disability among patients with type I and II BD have scarcely been studied. We investigated the clinical predictors of long-term work disability among patients with BD.

**Methods:**

The Jorvi Bipolar Study (JoBS) is a naturalistic prospective cohort study (n = 191) of adult psychiatric in- and out-patients with DSM-IV type I and II BD in three Finnish cities. Within JoBS we examined the prevalence and predictors of disability pension being granted during a six-year follow-up of the 152 patients in the labor force at baseline and collected information on granted pensions from national registers. We determined the predictors of disability pension using logistic regression models.

**Results:**

Over the 6 years, 44% of the patients belonging to the labor force at baseline were granted a disability pension. Older age; type I BD; comorbidity with generalized anxiety disorder, post-traumatic stress disorder or avoidant personality disorder; and duration of time with depressive or mixed symptoms predicted disability pensions. Including disability pensions granted before baseline increased their total prevalence to 55.5%. The observed predictors were similar.

**Conclusion:**

This regionally representative long-term prospective study found that about half of patients with type I or II bipolar disorder suffer from persistent work disability that leads to disability pension. In addition to the severity of the clinical course and type I bipolar disorder, the longitudinal accumulation of time depressed, psychiatric comorbidity, and older age predicted pensioning.

**Supplementary Information:**

The online version contains supplementary material available at 10.1186/s40345-022-00264-6.

## Background

Bipolar disorder (BD), once considered an illness with a good long-term outcome, has been estimated to be among the 20 leading causes of disability worldwide (Vos et al. 2012) Clinical outcome studies have shown that 30–60% of BD patients, even if in syndromic remission, are unable to attain social or occupational functioning (MacQueen et al. 2001). However, BD is an episodic and pleomorphic illness, and the psychosocial functioning of BD patients probably varies more than that of patients with any other psychiatric disorder. While the functioning of some patients is only temporarily impaired, and some even accomplish historical landmarks in human achievements, others experience significant long-term difficulties in managing even the tasks of daily living (Levy and Manove 2012).

### Bipolar disorder and functional ability

The predictors and mechanisms of disability in BD are still only partially known. Numerous demographic, clinical, and neurocognitive factors are associated with disability (Huxley and Baldessarini 2007; Sanchez-Moreno et al. 2009). Acute illness episodes strongly affect functioning, with the exception of hypomania (Judd et al. 2005; Simon et al. 2007). A major problem in BD is the recurrent nature of the illness, which involves multiple episodes over time. Even though most patients reach syndromal remission after an acute episode, almost all will also have at least one new episode in the following years (Gignac et al. 2015a, 2015b; Pallaskorpi et al. 2015). Besides the disrupting impact of multiple episodes, the length of time with symptoms is an often-overlooked factor that affects functional outcomes. Patients with BD-I or -II have been found to spend half of their time with symptoms in long term (Judd et al. 2002, 2003). Most of the symptomatic time involves subsyndromal depressive symptoms, but even modest changes in the severity of depression appear to be associated with changes in functional impairment and disability (Judd et al. 2005; Altshuler et al. 2006; Simon et al. 2007).

Unfortunately, many patients do not achieve their premorbid functioning even after reaching clinical remission (Sanchez-Moreno et al. 2009). The factors most consistently associated with functional impairment of patients with BD in remission are residual depressive symptoms and specific deficits in cognitive functioning (Judd et al. 2005; Altshuler et al. 2006; Rosa et al. 2009; Bonnin et al. 2010; Baune and Malhi 2015; Gitlin and Miklowitz 2017). However, although residual depression and cognitive deficits are important predictors of impairment, they explain only part of it (Bonnin et al. 2010). Therefore, it is important to investigate other possible predictors of functional disability (Schoeyen et al. 2013). Recent studies have found stressful life events, mood instability, impulsivity, inter-episode intensity and instability, and the level of personality functioning to be associated with functional impairment (Yan-Meier et al. 2011; Jimenez et al. 2012; Strejilevich et al. 2013; Gershon and Eidelman 2015; Kizilkurt et al. 2018).

The role of psychiatric comorbidity as a risk factor for disability among BD patients has been insufficiently investigated. In BD, comorbidity is the rule rather than exception (Goodwin and Jamison 2007), generally predicts a poor clinical outcome, and may reduce functioning even during remission from BD affective symptoms. Psychiatric comorbidities are common in BD, with anxiety disorders, substance abuse disorders, and personality disorders being the most prevalent. Using DSM-IV terminology, total Axis I lifetime comorbidity has been estimated to range from 35 to 80% (McElroy et al. 2001; Vieta et al. 2001; Simon et al. 2004; Mantere et al. 2006). The prevalence rates of personality disorder comorbidity among BD-I or II patients in euthymic state have ranged from 25 to 50% (Fan and Hassell 2008).

Anxiety disorders are the most prevalent comorbid diagnoses among BD patients. At least half of BD patients will suffer an anxiety disorder in their lifetime, and a third will manifest an anxiety disorder at any point of time (Spoorthy et al. 2019). Anxiety commonly covaries with depression (Mantere et al. 2010), but according to a recent meta-analysis, as much as 35% of euthymic BD patients have an anxiety disorder (Pavlova et al. 2017).

Overall, psychiatric comorbidity is associated with earlier onset of bipolar symptoms, greater functional and psychosocial impairment, poor adherence and response to treatment, prolonged recovery time, increased risk of suicide attempts and deaths, increased utilization of health services, and higher morbidity and mortality (Krishnan 2005; Lam et al. 2012). Even though comorbid disorders have been associated with many negative consequences in cross-sectional studies, long-term studies are scarce (Amann et al. 2017), and the impact of these on vocational abilities remains largely unknown.

### Bipolar disorder and long-term vocational disability

Work is an important part of functioning and a crucial contributor to the wellbeing and quality of life of BD patients (Sole et al. 2018). Measures of work impairment among BD patients vary and have even been found to be comparable to those among patients with schizophrenia (Dean et al. 2004). However, work disability is a multifaceted phenomenon with both short- and long-term perspectives being relevant. Most studies have focused on current work impairment in terms of occupational functioning, absenteeism and poor work performance, or long-term employment (Dean et al. 2004). Only a few cross-sectional studies (Gutierrez-Rojas et al. 2011; Grande et al. 2013; Schoeyen et al. 2013) have specifically investigated risk factors for long-term work disability or disability pension among BD patients. The predictors in these studies were the number of hospitalizations, illness duration, number of manic episodes, current depressive symptoms, Axis II comorbidity, having no stable partner, older age, and educational attainment.

### Aims of the study

In a previous medium-term follow-up study, we investigated the prevalence and predictors of disability pensions among BD patients (Arvilommi et al. 2015). During the 18-month follow-up, 25% of the patients were granted a new disability pension, predicted by both course of illness and comorbidity. However, the predictors of disability during and shortly after an acute episode may be different to the long-term predictors. Moreover, patients seek treatment for acute episodes, but the type of the index episode may strongly influence the findings in the short term, whereas long-term follow-up may reflect more general illness factors leading to disability pension.

Here, we present the data of the same patients as in our medium-term 18-month study, but with a five-year, long-term follow-up and register data of up to 6 years. As BD has a chronic nature, investigating the predictors of working disability over an extended period of time is essential (O’Donnell et al. 2017). We investigated the accumulation and predictors of long-term disability pensions.

## Methods

### Setting

The Jorvi Bipolar Study (JoBS) is a collaborative bipolar research project conducted by the Unit of Mental Health of the National Public Health Institute, Helsinki, and the Department of Psychiatry, Jorvi Hospital, Helsinki University Central Hospital (HUCH), Espoo, Finland. The Department of Psychiatry of the Jorvi Hospital provides secondary care in- and outpatient psychiatric services to all citizens of Espoo, Kauniainen, and Kirkkonummi (261,116 inhabitants in 2002). The Ethics Committee of HUCH approved the study protocol. The methodology of JoBS is described in detail elsewhere (Mantere et al. 2004; Pallaskorpi et al. 2015).

### Screening, diagnostic evaluation, and baseline measurements

Briefly, all inpatients and outpatients undergoing a current, possible new (DSM-IV) BD episode in the catchment area of Jorvi Hospital (N = 1630) were screened using the Mood Disorder Questionnaire (MDQ) (Hirschfeld et al. 2000) during the study period 1 January 2002 to 28 February 2003. After a positive MDQ screen or suspicion of BD (n = 546), the patients were fully informed of the study protocol and their written informed consent was requested. We made the BD diagnosis using the Structured Clinical Interview for DSM-IV Disorders (SCID-I/P) (First et al. 2002) and used all possible information, such as psychiatric records, interviews of family members, and the observations of attending personnel. The final sample consisted of 191 DSM-IV BD patients [90 BD-I (47.1%) and 101 BD-II (52.9%)] with a current episode (Mantere et al. 2004). To assess lifetime and current comorbid diagnoses we used the Structured Clinical Interviews for Axis I Disorders (SCID-I/P) (First et al. 2002) and Axis II Disorders (SCID-II) (First et al. 1997). To collect information on former illness history we used a retrospective life-chart. We also collected data on former and current suicidality, demographic characteristics, and treatments received.

**Fig. 1:**
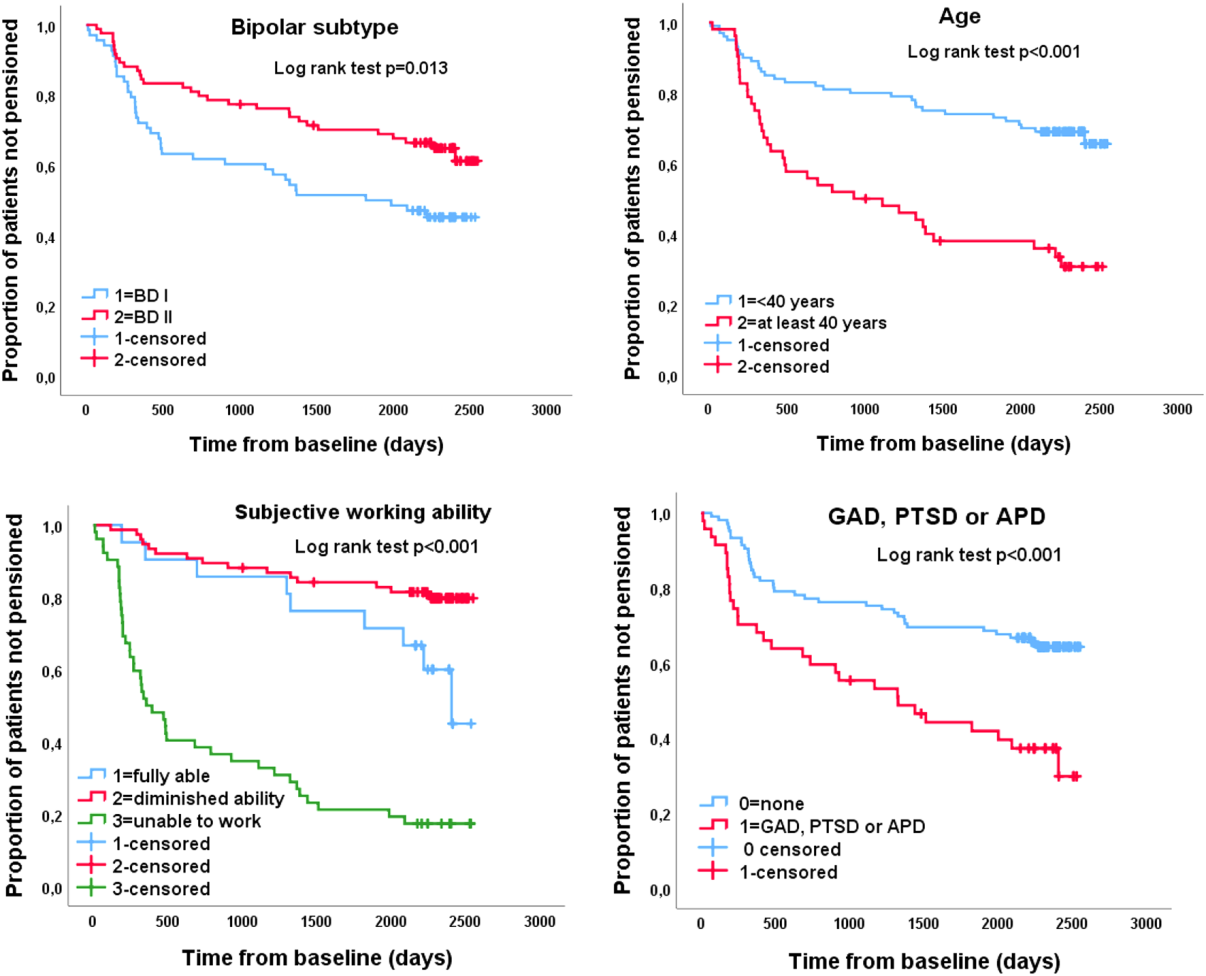
Kaplan–Meier survival curves of proportion of patients granted disability pension during 6-year follow-up of 152 patients not receiving pension at baseline

We used a range of observer and self-report scales, including Social and Occupational Functioning Assessment Scale of DSM-IV (SOFAS) (Goldman et al. 1992), Beck Depression Inventory (BDI) (Beck et al. 1961), 17-item Hamilton Rating Scale for Depression (HAM-D) (Hamilton 1960), Young Mania Rating Scale (YMRS) (Young et al. 1978), Beck Anxiety Inventory (BAI) (Beck et al. 1988), Perceived Social Support Scale-Revised (PSSS-R) (Blumenthal et al. 1987). We also assessed patients’ perceived work ability at baseline using an ordinal scale: 1 = fully able, 2 = diminished capacity, and 3 = unable to work. As the outcome for the first two alternatives did not differ (see Fig. 1), we combined them in multivariate analyses.

### Follow-up

We examined the outcome at 6 months, 18 months, and 5 years through repeated SCID-I/P interviews. We included all observer- and self-report scales in the follow-up assessments. All medical and psychiatric records were available. We then integrated all the data into the form of a graphic life chart based on DSM-IV criteria. The life chart only deviated from the DSM-IV by accepting hypomania of 2–3 days as hypomanic episodes, and defining the concept of a depressive mixed state, in line with Benazzi and Akiskal, as three or more simultaneous intra-episode hypomanic symptoms being present at least 50% of the time during a major depressive episode (Benazzi and Akiskal 2001). This approximates the current DSM-5 concept of major depressive episode with mixed features. Of the 191 patients undergoing a current illness phase initially included in the cohort, 176 were followed for 6 months, 168 for 18 months, and 112 for 5 years. In the analyses, all patients were treated according to their baseline SCID diagnosis.

### Disability pension

At intake, the participants consented to the collection of information on disability pensions granted during follow-up. This information was obtained from interviews, patient records, and the registers of the Social Insurance Institution of Finland and the Finnish Centre for Pensions. Information on disability pensions for the present study was obtained from the registers up to 31 October 2008 (from 5.52 to 6.98 years from baseline). The requirements for being granted disability in Finland are explained in Additional file 1. Although we had register-based information on all the pensions granted to the patients, for some patients we did not receive the exact dates and diagnoses related to the pension. In these cases, we collected this missing information from the medical records and from the interviews.

In the present study, all forms of disability pension, whether temporary or permanent, full time or part time, were treated as one group. Homemakers and individuals working part-time were considered to be working. Information on possible disability pensions from the registers was available for the whole JoBS cohort (N = 191). Patients who had already been granted a disability pension which had started before baseline and continued after it (n = 39, 20.4%) were excluded from the prospective analyses of the cohort follow-up because in their case the endpoint had already occurred. Thus, the analyses of the six-year follow-up included data on 152 [79.6%; 68 (44.7%) BD-I and 84 (55.3%) BD-II] of the 191 patients at baseline. During the follow-up, 8 of these 152 patients died (seven had been granted a disability pension before death and were therefore included in the analyses, and one who had not been granted a disability pension was included up to the time of death). The median follow-up period for disability pensions for the 152 patients was 6.04 years (from 11 days to 6.98 years). The last clinical follow-up interview in our study was at 5 years, so for some patients we had only register data from the last year of the 6-year follow-up.

### Statistical methods

First, we compared the sociodemographic and clinical characteristics of subjects who had been granted a disability pension and those who had not. The chi-square test was used as appropriate. Normally distributed continuous variables were analyzed using the two-sample t-test, and non-normally distributed variables were analyzed using the Mann–Whitney U-test. Second, we made logistic regression models to adjust for confounding factors and to determine the predictors of being granted a disability pension. The multivariate analyses were adjusted for age, gender, and BD subtype. All the hypothesized predictors and other variables that were significant or almost significant (p < 0.10) in the univariate analyses were added one by one to the multivariate analyses and included in the multivariate model if they were significant. To examine the effect of comorbidity, we included all the anxiety disorders and personality disorders in the multivariate analysis, irrespective of their significance in the univariate analyses. We made separate models by adding the time spent in different phases and proportions of the time spent in different phases during the follow-up. In these models we used information from the whole follow-up time, i.e., also phases after being granted a disability pension. Spearman bivariate correlations were computed to determine the inter-correlations between the predictors. We also used Kaplan–Meyer curves and log-rank tests to demonstrate subgroup differences. IBM SPSS statistics version 25 (SPSS Inc., Chicago, IL, USA) was used for the analyses.

## Results

### Patients granted a pension during the 6-year follow-up

Of the 152 patients followed, 67 (44.1%) were granted a disability pension. The primary clinical diagnosis (ICD-10) for being granted a disability pension was mainly BD (54/67, 80.6%). One or more auxiliary comorbid psychiatric diagnoses were recorded for 23 (15%) of the BD patients who were granted a disability pension.

### Sociodemographic and clinical differences

Already at baseline, the patients who were granted a disability pension during the follow-up differed in many respects from the non-pensioned patients (see Table 1). They were older, more often had BD-I, suffered from the disease for a longer period of time, had more depressive and manic episodes, and more often had alcohol use disorders. Whereas all the patients were in the acute phase at intake, those with later disability pension had lower levels of overall social and occupational functioning (assessed using the SOFAS), were subjectively (BDI) but not quite objectively (HAM-D) more depressed, perceived themselves as having less social support (PSSS-R), perceived their economic situation as worse, were more often on sick leave, and perceived themselves as unable to work considerably more often than their non-pensioned counterparts. No differences emerged in the proportion of pensioned and non-pensioned patients with anxiety disorders overall. However, as regards specific anxiety disorders, the pensioned patients more often had generalized anxiety disorder (GAD) and post-traumatic stress disorder (PTSD). The prevalence of patients with a personality disorder overall did not differ between these two groups, but as regards specific personality disorders, the pensioned patients more often had avoidant personality disorder (APD).

**Table 1:** Univariate analyses of predictors of work disability pension among employed patients with bipolar disorder in Jorvi Bipolar Study during 6-year follow-up

Predictor at entry	Being granted a disability pension during follow-up		
	Pensioned n(%)	Test statistic	p
Gender, male/female	37(51.4)/30(37.5)	χ^2^_1_ = 2.996	0.085
BD subtype, I/II	37(54.4)/30(37.5)	χ^2^_1_ = 5.330	0.021
Married or cohabiting, yes/no	27(43.5)/40(44.4)	χ^2^_1_ = 0.012	0.913
Living alone, yes/no	19(44.2)/48(44.0)	χ^2^_1_ = 0.000	0.987
Vocational education		χ^2^_3_ = 2.799	0.424
University	10(38.5)		
College	10(33.3)		
Vocational school	17(51.5)		
No professional education	30(47.6)		
Index phase depression, yes/no	41(49.4)/26(37.7)	χ^2^_1_ = 2.098	0.147
Psychotic symptoms LT, yes/no	35(47.3)/32(41.0)	χ^2^_1_ = 0.606	0.436
Alcohol dependence LT, yes/no	31(54.4)/36(37.9)	χ^2^_1_ = 3.931	0.047
Some anxiety disorder LT, yes/no	36(46.8)/31(41.3)	χ^2^_1_ = 0.453	0.501
Some personality disorder, yes/no	30(47.6)/37(41.6)	χ^2^_1_ = 0.547	0.460
Cluster A, yes/no	6(46.2)/37(43.9)	χ^2^_1_ = 0.025	0.875
Cluster B, yes/no	21(52.5)/46(41.1)	χ^2^_1_ = 1.562	0.211
Cluster C, yes/no	15(45.5)/52(43.7)	χ^2^_1_ = 0.032	0.857
Borderline personality disorder, yes/no	19(50.0)/48(42.1)	χ^2^_1_ = 0.721	0.396
Avoidant personality disorder, yes/no	11(68.8)/56(41.2)	χ^2^_1_ = 4.416	0.036
PTSD LT, yes/no	14(63.6)/56(40.8)	χ^2^_1_ = 3.992	0.046
GAD, yes/no	15(62.5)/52(40.6)	χ^2^_1_ = 3.923	0.048
Social phobia LT, yes/no	19(51.4)/48(41.7)	χ^2^_1_ = 1.049	0.306
Economic situation		χ^2^_3_ = 17,829	< 0.001
Good	2(10.5)		
Reasonable	18(35.3)		
Rather bad/poor	17(51.5)		
Bad/poor	28(63.6)		
Perceived work ability 1 + 2/3	24(24.7)/43(82.7)	χ^2^_1_ = 45.939	< 0.001
Fully able = 1	9(42.9)		
Diminished capacity = 2	15(19.7)		
Unable to work = 3	43(82.7)	χ^2^_2_ = 49.493	< 0.001
On sick leave at baseline, yes/no	44(62.0)/23(28.4)	χ^2^_1_ = 17.305	< 0.001

	Not pensioned mean (SD)	Pensioned mean (SD)	Mann–Whitney U	p
Age	32.1(10.17)	39.6(12.12)	3862.00	< 0.001
Duration of illness	10.62(8.58)	15.43(11.04)	3589.00	0.006
Number of episodes before baseline				
Depressive	7.09(11.56)	8.97(13.23)	3582.00	0.006
Manic	0.80(1.64)	2.33(3.77)	3449.00	0.006
Number of psychiatric hospital treatment periods	0.96(1.66)	1.75(3.01)	3257.00	0.090
SOFAS	52.88(10.35)	47.84(10.79)	1983.50	0.003
BDI	20.65(10.83)	27.03(11.34)	3689.50	< 0.001
HAM-D	17.03(7.44)	19.52(7.48)	3329.50	0.053
YMRS	7.40(8.14)	6.29(8.01)	2548.50	0.332
BAI	21.36(12.76)	24.15(12.00)	3205.50	0.142
PSSS-R	45.39(10.06)	38.61(13.73)	1955.00	0.003

*SOFAS* social and occupational functioning assessment scale, *BAI* Beck anxiety inventory, *BDI* Beck depression inventory, *HAM-D* Hamilton depression rating scale, *BHS* Beck hopelessness scale, *YMRS* Young mania rating scale, *PSSS-R* perceived social support scale-revised, *PTSD* post traumatic stress disorder, *GAD* generalized anxiety disorder, *LT* during lifetime

Perceived work ability is now under Economic situation in the pdf version

During the follow-up, the patients who had been granted a disability pension and those who had not did not differ in the proportions of patients who reached full remission (53/67 [79.1%] vs. 70/85 [82.4%], χ^2^ = 5.29, df = 1, p = 0.071). Neither was there a difference in the *number* of any of the phases. However, the proportion of *the total time spent in* euthymia was significantly smaller (mean 37.8% vs. 50.4%, Mann–Whitney U-test, U = 1950, p = 0.022), and the proportion of the time spent in major depressive phases (mean 37.6% vs. 22.0%, Mann–Whitney U-test, U = 3250, p = 0.003) and mixed phases (mean 2.0% vs. 0.3%, Mann–Whitney U-test, U = 2882, p = 0.014) was higher among the patients who were granted a disability pension than among the non-pensioned. The proportion of the time spent in the other phases did not differ.

Subjectively perceived inability to work at baseline was a strong predictor of being granted a disability pension during the follow-up (Fig. 1).

### Predictors of disability pension in multivariate logistic regression models

In the multivariate logistic regression models, being granted a disability pension was predicted by older age, BD-I, comorbidity with PTSD, and APD (See Table 2). As GAD correlated with PTSD (Spearman’s rho 0.283, p < 0.001) these could not be included in the model at the same time. GAD predicted being granted a disability pension (OR = 2.810, CI 1.014–7.784, p = 0.047) when included in the model instead of PTSD. When added to the model, perceived work ability was also a significant predictor (OR = 11.087, CI 4.450–27.623, p < 0.001), while all the other predictors, except age, lost their significance.

**Table 2:** Multivariate logistic regression model of predictors of disability pension during 6-year follow-up in Jorvi Bipolar Study

Predictor	Patients not on pension at baseline (N = 152)		
	OR	p	95% CI
Age	1.052	0.002	1.019–1.087
Sex	0.553	0.124	0.260–1.176
BD type I	2.610	0.012	1.240–5.491
PTSD lifetime	3.810	0.015	1.299–11.181
Avoidant personality disorder	4.357	0.022	1.234–15.383

When we included information from the follow-up in the model, we found that the proportion of time during the follow-up spent in depression (OR = 1.022, CI 1.006–1.038, p = 0.006), in euthymia (OR = 0.987, CI 0.974–0.999, p = 0.038), and in mixed phases (OR = 1.250, CI 1.023–1.526, p = 0.029) predicted being granted a pension, but PTSD was no longer significant. GAD was also no longer significant when included in the model instead of PTSD. In addition, APD was no longer significant when the proportion of time in depression was added. Note that the follow-up time included also time periods after being granted a disability pension.

### Patients who returned to work during the follow-up

During the six-year follow-up, 17 (25%) of the 67 patients who were in the labor force at baseline and on disability pension during the follow-up returned to work. Half (8/17, 47%) of these disability pensions lasted less than a year, and half (9/17, 53%) at least a year. Most (12/17, 71%) of the patients who returned to the labor force received no new disability pension periods during follow-up.

The patients who returned to the labor force were younger (only 1/35, 2.9% of patients aged > 40 at baseline returned vs. 16/32, 50% of patients aged ≤ 40 years, p < 0.001), less often had a professional education at baseline (4/17, 24%, vs. 33/50, 66%, p = 0.02), suffered from the illness for a shorter time (Mann–Whitney U-test p = 0.003), and had no history of panic disorder at baseline (0/16 patients with vs. 17/51, 33.3% without panic disorder, p = 0.008).

### Disability and return to work of all JoBS cohort patients

By the end of the 6-year follow-up, when we added the patients on disability pension already at baseline, altogether 106 (55.5%) of the original 191 patients had received a disability pension at some time point during the follow-up. Of the 106 patients, 19 (17.9%) returned to the labor force, 12 (11.3%) were still receiving temporary pension (five of them for at least one year), one died after three months and one after 6 months on disability pension, and 73 (68.9% of the 106, and 38.2% of the 191 patients) had been granted a permanent disability pension.

In the multivariate logistic regression models for the whole 191 patient cohort, being on disability pension at baseline or receiving a disability pension during the six-year follow-up associated with older age, BD-I, comorbidity with PTSD and APD (see Table 3). Note that for the patients who had been on disability pension already at baseline it is not known whether these clinical characteristics preceded being granted a disability pension.

**Table 3:** Multivariate logistic regression model of predictors of disability pension during 6-year follow-up for 191 patients in Jorvi Bipolar Study, including patients granted a disability pension before baseline

Predictor	All 191 patients in JoBS		
	OR	p	95% CI
Age	1.077	< 0.001	1.046–1.110
Sex	1.356	0.386	0.681–2.703
BD type I	2.483	0.009	1.258–4.902
PTSD lifetime	3.578	0.011	1.339–9.561
Avoidant personality disorder	4.149	0.019	1.267–13.594

## Discussion

We investigated the proportion and predictors of being granted a disability pension during a long-term follow-up among a representative cohort of patients with BD-I and BD-II. We found that nearly half of the patients belonging to the labor force at baseline were granted a disability pension during the 6-year follow-up. Being granted a disability pension was predicted by older age, BD-I subtype, and comorbidity with PTSD, GAD and avoidant personality disorder. Moreover, the total time spent in depression and mixed phases during follow-up were important predictors. In addition, subjective inability to work at baseline was found to be a strong predictor of future disability pension.

### Proportion of patients granted a disability pension

This study confirms the results of our 18-month study (Arvilommi et al. 2015) in that BD is a disabling illness, as nearly half (44%) of the BD-I and BD-II patients who were in the labor force were granted a disability pension during the 6-year follow-up. This is more than double the proportion of patients (20%) during a 5-year follow-up in a similar study of MDD patients in the neighboring city of Vantaa (Vantaa Depression Study, VDS) (Holma et al. 2012). Thus, BD seems to have a much worse vocational prognosis than unipolar MDD. This proportion is also nearly double that of the BD patients (25%) who were granted a disability pension in the first 18 months. Moreover, the survival curve did not flatten out by the end of the first 6 years, meaning that our findings may underestimate the accumulating lifetime prevalence. Including disability pensions granted before baseline increased their accumulated lifetime prevalence to 55.5%.

There are only a few previous studies that have reported the proportions of patients unable to work (Kogan et al.^ 2004^; Reed et al. 2010; Suppes et al. 2001) the proportions ranging from 15 to 22%. There are also very few studies reporting the proportions of patients with BD receiving a disability pension (Grande et al. 2013; Gutierrez-Rojas et al. 2011; Schoeyen et al. 2011a, b; Schoeyen et al. 2013), The proportion of patients with BD on a disability pension ranged in these studies from 17% among euthymic patients with BD (Grande et al. 2013) to 52.5% (also including patients who were in the process of receiving a disability pension) among patients selected from district computerized records as suffering from BD (Gutierrez-Rojas et al. 2011). Overall, the proportions of bipolar patients with a long-term disability found in the present study are in the upper limit of those in previous cross-sectional studies.

A positive finding was that, of the patients on long-term or permanent disability pension, sometimes for years, a quarter returned to the labor force during follow-up. Being less than 40 years old and suffering from the illness for a shorter time seemed to be the key predictors. Somewhat surprisingly, patients with a professional education returned more seldom, maybe due to the more demanding nature of their work. Interestingly, none of the patients with a history of panic disorder returned to the labor force during follow-up, even though panic disorder itself did not predict being granted a disability pension.

### Predictors of being granted a disability pension

As only a few previous cross-sectional studies have examined long-term work disability and disability pension among BD patients, the factors predicting these are not well-known.

#### Older age

Older age was the only strong independent sociodemographic predictor of being granted a disability pension. The risk of disability pension among patients aged over 40 was more than twice that among the younger patients. Age was also strongly associated with disability pension in our 18-month study, as in former studies (Grande et al. 2013; Schoeyen et al. 2013). Increasing age can affect the probability of being granted a disability pension in many ways. Older age is associated with longer duration and an accumulating burden of BD. However, age was significant even after adjusting for the duration of the illness, number of manic or depressive phases, and number of hospital treatment periods, so it also seems to have an effect independently of illness factors. Increasing age associates with an accumulating burden of physical illnesses and may also affect the way in which patients see their work ability, as age strongly correlated with subjectively perceived disability. The evaluating psychiatrists may also have a lower threshold for recommending disability pensions for older patients, and older patients may find pensioning more acceptable.

#### Bipolar subtype

We confirmed our medium-term finding, that BD-I patients are granted a disability pension more often than BD-II patients. BD-II may (Serra et al. 2017) or may not (Joffe et al. 2004; Kupka et al. 2007; Pallaskorpi et al. 2015) have a more chronic course than BD-I, with more time spent with depressive symptoms than in cases of BD-I (Serra et al. 2017). Our results are in line with the study by Judd et al. (Judd et al. 2008) finding that BD-I patients were unable to carry out work role functions for significantly greater proportion of time than BD-II patients. We found that BD-I patients had more hospital treatment periods before baseline and were more often treated in hospital during the index phase, as other studies have also found (Vieta et al. 1997; Rosa et al. 2010). This may indicate that symptom severity is greater among BD-I patients, which may partly explain the more frequent granting of disability pension. The disruptive effect of recurrent manic phases in particular may be a considerable threat for vocational careers.

#### Depression

We confirmed that also in the long term, disability pensions were associated with more time spent in major depressive episodes during follow-up. Former studies have also found current depression, either syndromal or subsyndromal, to be one of the most consistent predictors of work ability (Huxley and Baldessarini 2007; Sanchez-Moreno et al. 2009). The number of previous episodes has also been reported to predict functional disability, but it remains unclear whether previous manic or previous depressive phases have the more deleterious effect (Grande et al. 2013). Former studies with disability pension as an outcome measure have reported somewhat discrepant findings regarding the impact of current and previous episodes on disability (Gutierrez-Rojas et al. 2011; Grande et al. 2013; Schoeyen et al. 2013). It appears that the effect of depression on vocational disability is more concurrent and related to chronicity (Gutierrez-Rojas et al. 2011), whereas the disruptive effects of mania on professional careers may accumulate with a progressing number of episodes (Gutierrez-Rojas et al. 2011; Grande et al. 2013).

#### Comorbidity

Even though *c*omorbidity with anxiety disorder or personality disorder did not predict disability pensions overall in the long term, we found that comorbidity with specific anxiety and personality disorders, including GAD, PTSD, or APD, did. This is in line with our former 18-month study, in which we found that GAD and APD specifically predicted disability pension in the medium term. In this long-term study in which more patients were granted a disability pension, the effect of some specific comorbid disorders appeared stronger. Our findings are not surprising in the light of former studies, as comorbidity with anxiety and personality disorder have been associated with many negative aspects of the course and outcome of the illness (Fan and Hassell 2008; Spoorthy et al. 2019). However, there are very few prospective studies of the impact of comorbid anxiety or personality disorders on the long-term course of BD (Coryell et al. 2009, 2012; Kim et al. 2014; Amann et al. 2017; Serra et al. 2017; Post et al. 2020). The impact of these comorbidities on long-term work disability or disability pension has been studied even more rarely. In their cross-sectional study, Grande et al. (2013) found that Axis II was associated with receiving severe disability benefit at the time of the study, but they did not report the significance of specific personality disorders. They found no significant association between anxiety disorder comorbidity and disability pension, nor did they report results concerning specific anxiety disorders.

One possible explanation for our findings is that patients with these comorbidities spend a greater proportion of time symptomatic and specially in depressive phases (Coryell et al. 2009, 2012; Kim et al. 2014; Amann et al. 2017; Serra et al. 2017). In line with this, we found that patients with comorbid GAD and PTSD spent more time in depressive and mixed depressive phases, and less time in euthymic phases, and patients with APD in depressive phases during the follow-up than patients without these comorbidities. In addition, when we added the proportions of time spent in the depressive, mixed or euthymic phases during the follow-up to the regression model, PTSD and GAD were no more significant, as also APD when the proportion of time in spent in depression was added to the model. So, it may be that the effect of PSTD, GAD and APD on disability pension is partly mediated by affecting the time spent in these phases. This is in line with studies by Coryell et al. (2009, 2012) and Serra et al. (2017).

A key similarity between comorbidities with GAD, PTSD, and APD is the persistence of their symptoms during periods of euthymia (Bennett et al. 2019). Thus, for patients with either of these disorders, symptoms of negative emotionality, worry, and tension are likely to persist into the euthymic periods (Boylan et al. 2004), which also negatively impacts functioning. Some patients may work well despite subsyndromal symptoms, but patients with these comorbid disorders may function poorly even when their mood symptoms do not reach the level of an episode.

Although our results that comorbidities are important predictors of long-term work disability may still be considered preliminary, possible implications of treatment need to be noted, as treatments for them differ from those for BD. After the acute phase of BD has been treated, possible comorbidities should be diagnosed, and treatment needs should be considered. Too often the treatment of BD focuses only on the treatment of the acute mood phases. However, current guidelines offer little help for clinicians in managing these disorders with BD (Bennett et al. 2019).

#### Subjective work ability

As in our 18-month study, patients’ subjectively perceived work ability at baseline was a strong predictor of a disability pension during the six-year follow-up. Of patients who perceived themselves as fully unable to work five sixths (83%) were eventually granted a disability pension, but only a quarter (25%) of those who felt at least partly capable. This is also in line with the findings of the five-year follow-up of unipolar MDD patients in the VDS (Holma et al. 2012). Perceived poor or lacking work ability correlated with age, duration of illness, number of hospital treatment periods, SOFAS, a depressive index phase and a greater proportion of time spent in depressive or mixed/mixed depressive phases during the follow-up, and thus relates to a more difficult and depressive course of BD. Thus, in addition to an individual patient’s experience, their subjective perception appears to be firmly rooted in the severity of the illness and the clinician’s assessment of the level of functioning. This is in line with the study of Karpov et al. (2017), which found that perceived and actual work ability correlated among mood disorder patients. However, in addition to actual work ability, perceived work ability is likely to include other subjective aspects of vocational ability. Perceived cognitive ability has been associated with work motivation (Martinez-Camarillo et al. 2019) and may be one explanation for the strong impact of subjectively perceived work ability on being granted a disability pension. Thus, perceived inability to work is in itself likely a subjective outcome of multiple factors. We nevertheless find it of clinical interest, how strongly such a subjective estimate of ability to work at one point in time predicts being granted a disability pension during the following years.

### Strengths and limitations

To the best of our knowledge, not including our 18-month study, this is the first prospective long-term study of predictors of disability pension among BD-I and II patients. The strengths of the JoBS include a relatively large clinical cohort from community-level psychiatric care, with a catchment area of three Finnish cities, and systematic screening for BD using the MDQ (cut-off modified to maximize sensitivity) among both psychiatric inpatients and outpatients. The study also comprised patients with all kinds of index phases and types of BD, inpatients and outpatients, and patients both with and without a clinical BD diagnosis at baseline. The patients were followed up for five years and register data on disability pensions for up to 6 years. The use of prospective life-chart methodology allowed us to analyze the influence of the accumulating time spent in different types of syndromal and subsyndromal states as a predictor of disability pension. The JoBS patients were carefully diagnosed on the basis of semi-structured interviews, with excellent reliability for both BD-I and BD-II. In addition, Axis I and II comorbid disorders were assessed using the SCID-I/P and SCID-II. We used register-based data to get precise information of all the patients on the granted disability pensions and when they were granted, and of hospital treatments, their dates and diagnoses. We were able to investigate a wide range of possible predictors, including factors related to BD, comorbid disorders, sociodemographic and psychosocial factors, and dimensions of personality.

However, some methodological points need to be addressed. First, it is important to bear in mind that the endpoint of interest in this study was disability pension granted because of long-term disability due to BD. This is different from investigating the level of overall functioning, employment outcomes, short-term absenteeism or sick-leaves, or poor functioning at work. As the prerequisite for being granted a disability pension is that the person has been on sick leave for 300 days in the preceding two years, the time of work disability started before baseline for some patients. Second, when we included information of proportions of time spent in different phases during the follow-up in the logistic regression model, we used information from the whole follow-up time, i.e., also phases after being granted a disability pension. We used these proportions of time as indicators of impact of longitudinal illness course on long-term disability. Although being granted a disability pension is a discrete event, it is preceded by 300 days of sickness absence, and the temporal course of disability is actually ambiguous and intertwined with course of illness. Third, when we used logistic regression model for the whole 191 patients, for some of the patients some of the associated characteristic may have been present only after being granted a disability pension. Fourth, this study was naturalistic, and the influence of the treatment on outcome could not be controlled. Fifth, we lacked in-depth information on the influence of comorbid somatic illnesses on work ability. Sixth, the number of patients reaching the endpoint was small in some subgroup analyses, rendering them vulnerable to type II errors. Therefore, the findings of the subgroup analyses must be interpreted with caution. Seventh, we did not measure cognitive functioning, which may considerably influence functional ability, even in euthymic BD patients (Bonnin et al. 2010; Baune and Malhi 2015). Eighth, the reliability of the diagnoses of comorbid mental disorders or the life chart data were not formally tested. Ninth, the study data were collected over ten years ago. Nevertheless, as the conditions on the basis of which a disability pension can be granted have not significantly changed in Finland since the beginning of the study, we consider this limitation somewhat theoretical. The only significant epidemiological change has been the increased number of BD diagnoses, likely due to improved recognition; but this in itself is unlikely to greatly influence the predictors of disability pension. Finally, although our study population is a representative sample of secondary care psychiatric patients in Finland, whether our findings can be generalized to other psychiatric settings, or to other health and social insurance systems with possibly different criteria for evaluating work ability, remains unknown.

### Conclusions

BD is associated with a major risk of long-term work disability, as about half of BD patients are granted a disability pension within six years of an acute phase. In addition to age, BD subtype, and the severity of the clinical course, comorbidity is a main predictor. Longitudinally, the accumulation of the depressive burden is also fundamental. In addition to adequate treatment of affective episodes, the diagnosis and treatment of comorbid disorders should receive more attention.

## Supplementary Information

**Additional file 1. **Disability pensions in Finland.

## Data Availability

Data not publicly available due to restrictions imposed by the Finnish legislation and patient consents.

## Abbreviations

APD: Avoidant personality disorder
BD: Bipolar disorder
BD-I: Bipolar disorder type I
BD-II: Bipolar disorder type II
GAD: Generalized anxiety disorder
JoBS: The Jorvi Bipolar Study
MDD: Major depressive disorder
MDQ: Mood disorder questionnaire
PTSD: Post-traumatic stress disorder
SCID-I/P: The Structured Clinical Interviews for Axis I Disorders
SCID-II: The Structured Clinical Interviews for Axis II Disorders
SOFAS: Social and occupational functioning assessment scale
VDS: Vantaa Depression Study
