# Supplementary material for: Long-term work disability due to type I and II bipolar disorder: findings of a six-year prospective study
Source: Int J Bipolar Disord. 2022 Jul 11;10:19. doi: 10.1186/s40345-022-00264-6 (PMC9271449; doi:10.1186/s40345-022-00264-6)
Supplement: Supplementary file 1 — Additional file 1. Disability pensions in Finland. [file 40345_2022_264_MOESM1_ESM.docx]

**Additional file 1.**

**Disability pensions in Finland**

In Finland, employees aged under 63–65 years become eligible for a disability pension, if after first having received daily allowance from sickness insurance for 300 days during a two-year period (counted at six days per week; the 300 days usually comprises several consecutive shorter sick leave periods) are still considered unable to work because of an illness.

Medical certiﬁcates issued by a psychiatrist for work disability allowances are referred to and granted by the Social Insurance Institution of Finland and by other pension providers; records on all pensions granted in Finland are collected by the Finnish Centre for Pensions. The presence of an illness is a necessary precondition for a disability pension to be granted, but the decision is made based on patients’ current and expected future capacity for functioning. The basic information for this decision is from the attending clinicians report in the medical certiﬁcate. Thus, being granted a disability pension indicates true long-term work disability due to an illness.

The pension is usually ﬁrst temporary, sometimes for several consecutive years, but a permanent disability pension is granted when the likelihood of regaining the ability to work is low. A part-time pension may also be granted.

*Modified from: Arvilommi P, Suominen K, Mantere O, Valtonen H, Leppämäki S, Isometsä E. Predictors of long-term work disability among patients with type I and II bipolar disorder: a prospective 18-month follow-up study. Bipolar Disord 2015; 17: 821-835.*
